# The role of healthy dog carriers of *Babesia microti*-like piroplasms

**DOI:** 10.1186/s13071-019-3371-5

**Published:** 2019-03-26

**Authors:** Rocío Checa, Luis Eusebio Fidalgo, Ana Montoya, Ana María López, Juan Pedro Barrera, Rosa Gálvez, Sara Sánchez de la Nieta, Valentina Marino, Juliana Sarquis, Guadalupe Miró

**Affiliations:** 1 Department of Animal Health, Veterinary Faculty, Universidad Complutense de Madrid, Madrid, Spain; 2 Department of Veterinary Clinical Sciences, Veterinary Faculty, Universidad de Santiago de Compostela, Lugo, Spain

**Keywords:** *Babesia microti*-like piroplasm, *Babesia canis*, Healthy dog carriers, Northwestern Spain, Canine piroplasmosis

## Abstract

**Background:**

While in Europe *Babesia canis* has been traditionally held responsible for canine piroplasmosis, *Babesia microti*-like piroplasm (Bml) infection is being ever more observed in dogs, with the first clinical cases reported in northwestern Spain. This study examines the epidemiological role of healthy dogs living in endemic areas of Bml infection in Spain. The data obtained were used to describe the clinical status and map the geographical distribution of Bml infection in healthy dogs in northwestern Spain.

**Results:**

Blood samples and ticks were taken from 756 healthy dogs representatively across the whole Galicia region (northwestern Spain): stray (*n* = 211), hunting dogs (*n* = 333) and pets (*n* = 212). Blood samples were tested by microscopy parasite observation, nested PCR-RFLP and sequencing. Piroplasm infection prevalences in healthy dogs from northwestern Spain were 17.1% (129/756) by PCR and 3.4% (26/756) by microscopy observation. The species found by PCR were: 2.2% (17/756) for *B. canis* and 15.1% (114/756) for Bml. Co-infection with *B. canis* and Bml was noted in 2 dogs. The higher prevalences detected were Bml in hunting dogs (25.5%; 85/333) and *B. canis* in stray dogs (6.6%; 14/211). In fox-hunting dogs from any area and dogs from the A Coruña Province, significantly higher prevalences of Bml infection were detected (*P *< 0.001). Upon physical examination, tick infestation was observed: 130 ticks in 18 hunting and three pet dogs. These were subsequently identified as *Rhipicephalus sanguineus* (*s.l.*) (49.2%), *Ixodes hexagonus* (38.5%), *Ixodes ricinus* (6.9%) and *Dermacentor reticulatus* (5.4%). Among the more prevalent ticks infesting healthy carrier dogs were *I. hexagonus*, followed by *D. reticulatus* and *I. ricinus*.

**Conclusions:**

*Babesia canis* and Bml were the only piroplasm species found infecting healthy dogs in Galicia, the prevalence of Bml being higher than of *B. canis*. Factors correlated with a higher Bml infection risk were being a hunting dog and living in the A Coruña Province. Healthy dogs travelling to other countries could act as carriers and probably contribute to the spread of Bml infection in dogs and wild carnivores throughout Europe.

## Background

Canine piroplasmosis is a worldwide severe tick-borne hemoprotozoan disease caused by several species of the genera *Babesia* and *Theileria* [1]. Based on the morphology of the merozoites infecting erythrocytes, these parasites are classified as large (3–5 μm) or small piroplasms (0.5–2.5 μm) [1]. Both large (*Babesia canis* and *Babesia vogeli*) and small merozoites of *Babesia* species (*Babesia gibsoni* and *Babesia microti*-like isolates also referred to as *Babesia vulpes* or “Theileria annae”) infect dogs in Europe [2].

Traditionally, endemic areas of canine piroplasmosis in Europe have been related to the distribution of its tick vectors [3]. *Babesia canis* is transmitted by *Dermacentor* spp. and is the predominant piroplasm species reported in Europe (from Portugal to the north and east of Europe) with a higher prevalence in central Europe [2]. *Babesia vogeli* is associated with the distribution of the brown dog tick *Rhipicephalus sanguineus*, restricted to the Mediterranean basin while the small piroplasm *B. gibsoni* is only sporadically found in Europe, mostly when infected dogs are imported from endemic areas (Asia, USA and Australia) [4]. It has been proposed that *B. gibsoni* could be transmitted by *R. sanguineus* (*s.l.*) in much of tropical Asia or Europe, but there are still no data to confirm this hypothesis. In Japan, *B. gibsoni* is endemic and is naturally transmitted by *Haemaphysalis* tick species [1]. Direct dog-to-dog transmission through fighting is the major route of *B. gibsoni* infection in American Pit Bull Terriers and related breeds in which it is more prevalent, and this could be the main mode of transmission outside endemic regions [5].

The other small piroplasm species reported in Europe is *Babesia microti*-like sp. (Bml), which was first detected in 2000 in a dog in Germany that had travelled to northwestern Spain in 1994 [6]. In later studies by Camacho et al. [7] and García et al. [8], this new pathogen was detected in several dogs in northwestern Spain, where canine piroplamosis has been traditionally caused by the large piroplasm *Babesia canis*. However, individual clinical reports of Bml in dogs in Europe are increasing. Furthermore, using molecular methods, this small piroplasm has been identified in dogs in Spanish regions outside Galicia such as Barcelona and Asturias [4, 9], although the travelling history of positive dogs is unknown. Reports also exist for other European countries including Portugal [10], Croatia [11], Sweden [12], France [13] and Serbia [14], suggesting that canine piroplasmosis caused by Bml is more frequent than was previously thought. To date, however, few studies have provided data on the prevalence of this piroplasm infection in dogs across Europe. So far, higher prevalences of Bml infection have been reported in red foxes (*Vulpes vulpes*) in northwestern Spain and Portugal, while *B. canis* has been only rarely identified in these wild carnivores [15, 16]. The infection of red foxes by Bml has also been reported in central and northern Spain [17–19], Croatia [20], Italy [21], Hungary [22], Great Britain [23], Slovakia [24], Germany [25], Austria [26], Bosnia and Herzegovina [27] and Israel [28]. In effect, some of these European countries have reported Bml infection in foxes but not in dogs. Outside Europe, Bml has been detected in one fox in Canada and in red foxes and fighting dogs in the USA [5, 29, 30]. The high prevalence of Bml infection found in red fox populations suggests they could be the main reservoir of Bml both in Europe and the USA. Currently, the literature lacks data regarding the clinical impact of Bml in foxes [4]. Presently, the only recognized endemic area of Bml infection in Europe is Galicia. This large region is more suitable than other Spanish regions for the proliferation of foxes (with the highest densities of 5.6 foxes/km^2^) [31] probably owing to the abundance of rural and peri-urban habitats such as farms or uncontrolled rubbish dumps which act as food sources for foxes.

Dogs infested by ticks or those more exposed to ticks such as hunting dogs, sheep dogs or dogs living outdoors are especially vulnerable to *Babesia* spp. infection [4]. Although the transmission vector of Bml is presently unknown, the species *Ixodes hexagonus* or “the hedgehog tick”, as is commonly known, has been suggested as the main tick vector since *I. hexagonus* has been found more frequently than other ticks in Bml-infected dogs [32]. Furthermore, this tick species is the one most frequently detected in wild carnivores such as red foxes [15]. However, other tick species could be involved in the transmission cycle of this protozoan [10, 15, 33], as Bml DNA has also been detected in *I. ricinus*, *I. canisuga*, *R. sanguineus* (*s.l.*) and *Dermacentor reticulatus* [34–36]. These candidate vectors could explain the detection of Bml in domestics and wild canids in areas lacking *I. hexagonus* [12, 29], though their competence as vectors for Bml has yet to be confirmed. Finally, other non-vector routes of transmission that may be involved are blood transfusion, vertical or dog-to-dog.

Most reports of Bml infection in dogs in Europe have described individual clinical cases of acute or peracute canine babesiosis. Epidemiological data on the prevalence of clinical illness or subclinical infection are scarce [2]. This study was conducted in the main endemic region of canine piroplasmosis in Europe. Bml infection was identified in several apparently healthy dog populations with different lifestyles and geographical distributions. Our objective was to address the epidemiological role of healthy dog carriers of Bml piroplasm by determining prevalences and associated risk factors. The resultant data were used to describe the clinical status of Bml infection and to generate a distribution map for the study area.

## Methods

### Study design

#### Sample size

The sample size needed to determine prevalence was estimated using the winepi.net programme for a confidence interval of 99% and desired absolute precision of 2%. The following epidemiological data were considered: general dog population in Galicia and an expected prevalence of 1.9% for Bml according to the results of a previous study carried out on the dog population of northwestern Spain [37].

#### Dog population

The animals surveyed (*n* = 756) in this cross-sectional study were classified based on their lifestyle as previously described by Miró et al [38]. The first group comprised high-risk outdoor dogs: stray dogs (*n* = 211) abandoned in any Galicia region re-homed in animal shelters until their adoption; and hunting dogs (*n* = 333) living in small kennels (two or four per kennel) close to their owners’ houses and potentially in close contact with wild animals during their hunting activities. The stray dogs sampled were subjected to a health programme in the shelters while most of the hunting dogs sampled had not followed a proper control programme including ectoparasiticides. These stray and hunting dogs were assumed to be highly exposed to ticks or prone to fighting because of their outdoor lifestyle, overpopulation and stress. The second group comprised low-risk dogs: pets (*n* = 212). These owned healthy dogs, recruited during yearly rabies vaccination and parasite checks, were considered to carry a lower infection risk.

#### Inclusion criteria

Inclusion criteria for the enrolment of dogs were apparently healthy dogs of any breed or sex and age, not showing clinical signs suggestive of acute canine piroplasmosis such as severe pale mucous membranes, apathy, anorexia, fever, jaundice and/or pigmenturia or pigmented faeces (indicating bilirubin excretion).

### Study area

Dogs were enrolled from the four provinces of the Galicia region (northwestern Spain): A Coruña (*n* = 285), Lugo (*n* = 165), Ourense (*n* = 136) and Pontevedra (*n* = 170). The climate of this region is oceanic-humid determining warm summers, cool winters and rain throughout the year.

**Fig. 1:**
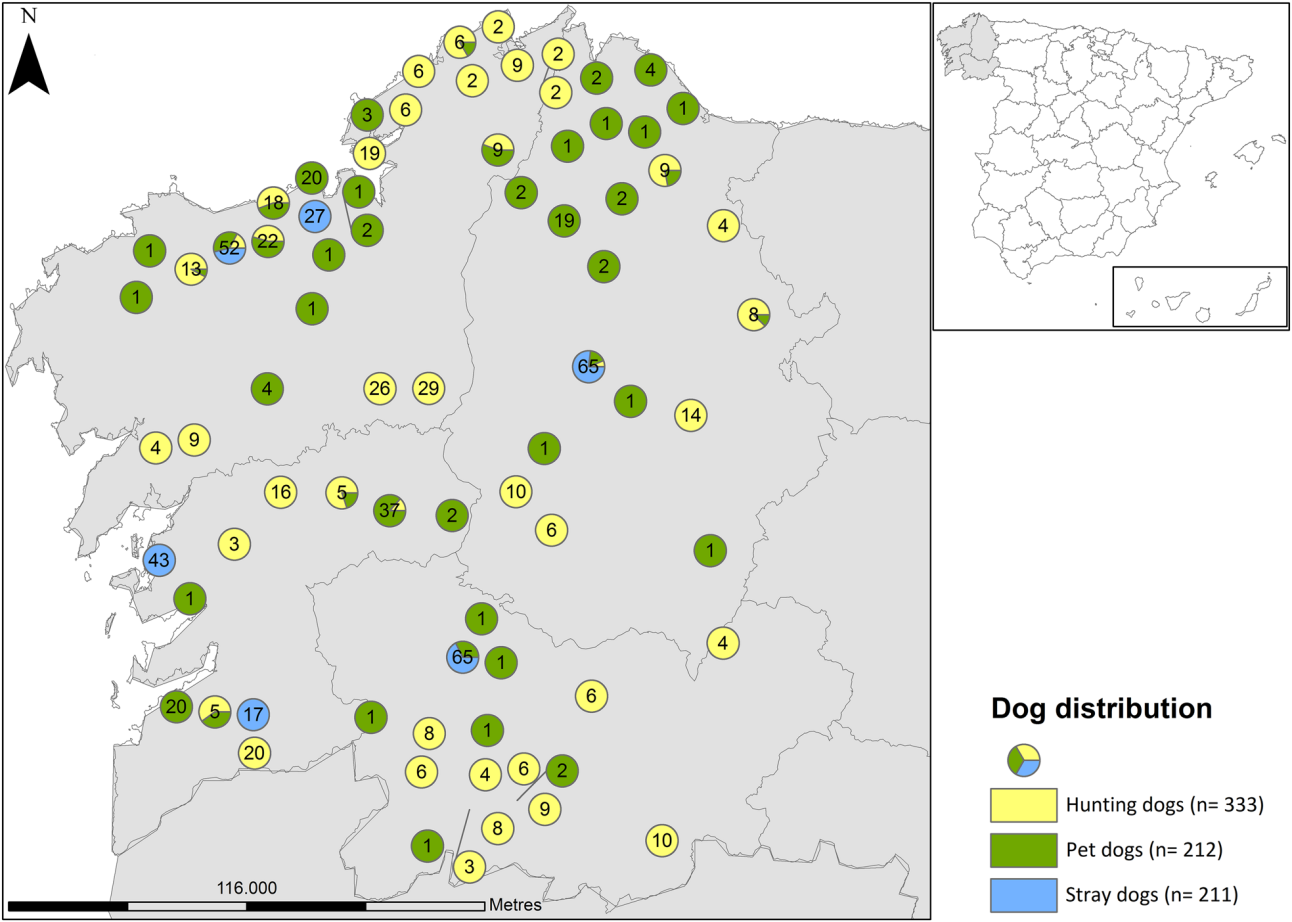
Sampling sites in northwestern Spain

The stray dogs examined were housed in kennels at six shelters belonging to different animal protection organizations of the four Galician provinces: two shelters in the A Coruña Province (at Carballo and Culleredo), two in Pontevedra Province (at Cambados and Ponteareas), one in the Lugo Province (Lugo) and the other in the Ourense Province (Ourense). Stray dogs had been sterilized under a health control programme. The hunting dogs examined lived in small kennels (two or four dogs per kennel) close to their owners’ houses. These dogs were from 35 representative locations across the Galician regions. Pet dogs were examined at 12 veterinary clinics across Galicia. These were companion dogs that lived in flats or houses in urban or rural areas of Galicia (Fig. 1).

### Sample and data collection

#### Prevalence survey

Over a one-year period (August 2017 to August 2018), 756 healthy dogs across the three risk groups (stray, hunting and pet) were recruited. All dogs underwent a thorough physical exam and blood collection by cephalic venipuncture. Blood was collected (2 ml per dog) into two tubes containing EDTA (1 ml) for parasite detection through DNA isolation, nested PCR and sequencing. Blood smears were prepared and fixed in methanol immediately after blood collection. Blood samples mixed with EDTA were kept initially at 4 °C and later stored at -20 °C until molecular processing in the laboratory.

The following data were collected in a clinical record: date, origin, age, breed, sex, weight, lifestyle, ectoparasites and a brief clinical history.

#### Bml-infected dogs

Once the diagnostic procedures described above had been carried out, owners were contacted (within a week) and a further blood sample was drawn (2.5 ml) from dogs testing positive for Bml: 0.5 ml were placed in an EDTA tube for full blood counts and 2 ml in tubes without anticoagulant for biochemical profiles.

Complete blood counts (CBC) consisting of leukocyte count (WBC), red blood cell count (RBC), haematocrit, haemoglobin concentration, red cell distribution width (RDW), mean corpuscular volume (MCV), mean corpuscular haemoglobin (MCH), mean corpuscular haemoglobin concentration (MCHC) and platelet count. Biochemical profiles included total serum protein, urea, creatinine, aspartate aminotransferase (AST), alanine aminotransferase (ALT) and symmetric dimethylarginine (SDMA).

### Parasite detection

#### Microscopy

Thin blood smears were Diff-Quick stained and examined by light microscopy using a 1000× magnification objective under immersion oil to detect intraerythrocytic ring-shaped bodies compatible with piroplasm merozoites.

#### DNA purification from blood

DNA from peripheral whole blood samples was isolated and extracted using QIAamp® DNA mini kit (Qiagen, Hilden, Germany). Briefly, 200 μl of each blood sample and 20 μl of proteinase K were added to a 1.5 ml tube with 200 μl of AL buffer (included in the kit) and incubated at 56 °C for 10 min. Subsequent steps were performed according to the manufacturer’s instructions (QIAamp® DNA mini and blood mini handbook provided by the manufacturer). Finally, the extracted DNA was eluted in sterilized water (200 μl) and stored at -20 °C until further use.

#### Molecular analysis

Blood DNA samples were tested using two nested PCR methods targeting the *18S* rRNA gene. *Babesia*/*Theileria* genus-specific nested PCR-based assays were performed using primer sets BTF1/BTR1 and BTF2/BTR2, which have been shown to be sensitive for the detection of piroplasms in dog blood [39]. To discriminate between species within the piroplasm-positive samples detected by nested PCR, a restriction fragment length polymorphism method (RFLP) was used on the PCR products of the second round of amplification (800 bp) using *Taq*I and *Hinf*I enzymes as described by René-Martellet et al. [13].

Specific nested PCR for Bml detection was carried out employing the universal BT1-F/BTH-1R *Babesia* and *Theileria* primers and the specific primers BTFox1F/R Bml, yielding a 655 bp fragment [23]. The reaction mixture was prepared as described elsewhere (see Checa et al. [15]).

Restricted fragments were examined by electrophoresis on a 2% agarose gel and 15 μl and 10 μl of the PCR products for the first and second PCR-based essay, respectively, were run on a 1.5% agarose gel stained with SYBR® Safe Gel Stain (Invitrogen, Waltham, USA) and visualized under UV light. All PCR protocols have been previously validated in our laboratory.

#### DNA sequencing

PCR products were purified using the QIAquickGel® extraction kit (Qiagen). Products corresponding to the expected length were excised with a clean scalpel from the agarose gel and kept in three volumes of QG solution (included in the kit) in a 1.5 ml tube. After mixing and dissolving the gel fragment at 50 °C for 10 min, the samples were added to the column (provided by the manufacturer) and centrifuged as described by the manufacturer. In the last step, the purified DNA was incubated for 1 min at room temperature and eluted in 30 μl of sterilized water.

The products were sequenced with the corresponding PCR primer set (BTF2/BTR2 or BTFox1F/R) at the Genome Sequencing Service (Universidad Complutense de Madrid, Madrid, Spain) using an ABI Prism 3730 (Applied Biosystems, Foster City, USA). The sequence chromatogram files obtained were edited, assembled and aligned using Chromas v.2.1.1 with BioEdit v.7.0.5 software. Edited DNA sequences were compared to those available in GenBank using the BLAST program.

Samples returning positive PCR-RFLP results were submitted for sequencing with BT2F/R primers. All Bml-positive (PCR-RFLP) sequences showed 99–100% homology with some *B. microti*-like isolates (GenBank: KT223483.1, AY534602.1, EU583387.1) while *B. canis*-positive sequences displayed 99–100% similarity with some isolates of *B. canis* (GenBank: KY747491.1, KC593877.1). Additionally, two samples yielding positive *B. canis* PCR-RFLP results that tested Bml-specific PCR positive underwent sequencing with BTFox1F/R primers. These two sequences showed 99–100% similarity with isolates of *B. microti*-like piroplasm (“Babesia annae” and *Babesia* “Spanish dog isolate”, GenBank: KT580785.1 and EU583387.1, respectively).

### Statistical analysis

All statistical tests were performed using the package IBM SPSS Statistics version 25.0 (IBM, New York, USA). A descriptive analysis was performed using standard statistics for qualitative variables (absolute and relative frequencies) and quantitative variables (mean and standard deviation). Microscopy results were compared with molecular results using the simple kappa coefficient. Relationships between Bml infection and the remaining categorical variables were assessed using the Chi-square test and between Bml infection and continuous variables by the Wilconson rank-sum test. To construct the decision tree model, we chose predictors according to their statistical significance, thereby allowing us to detect any interactions with the variable Bml-infected dog. For predictor variables, this method determines the optimal cut-off that maximizes the association with the target variable. It provides highly interpretable results and served to identify groups of infected dogs with homogeneous behaviour in the response variable (Bml-infected dog). Significance was set at *P *< 0.05.

### Tick identification

After clinical examination, ticks collected from each dog were kept in individual vials containing 70% ethanol. Ticks were identified at our parasitology laboratory to the species level, sexed and their stage (larvae, nymph or adult) determined using morphological keys [40–42].

## Results

### Molecular and sequencing results

**Fig. 2:**
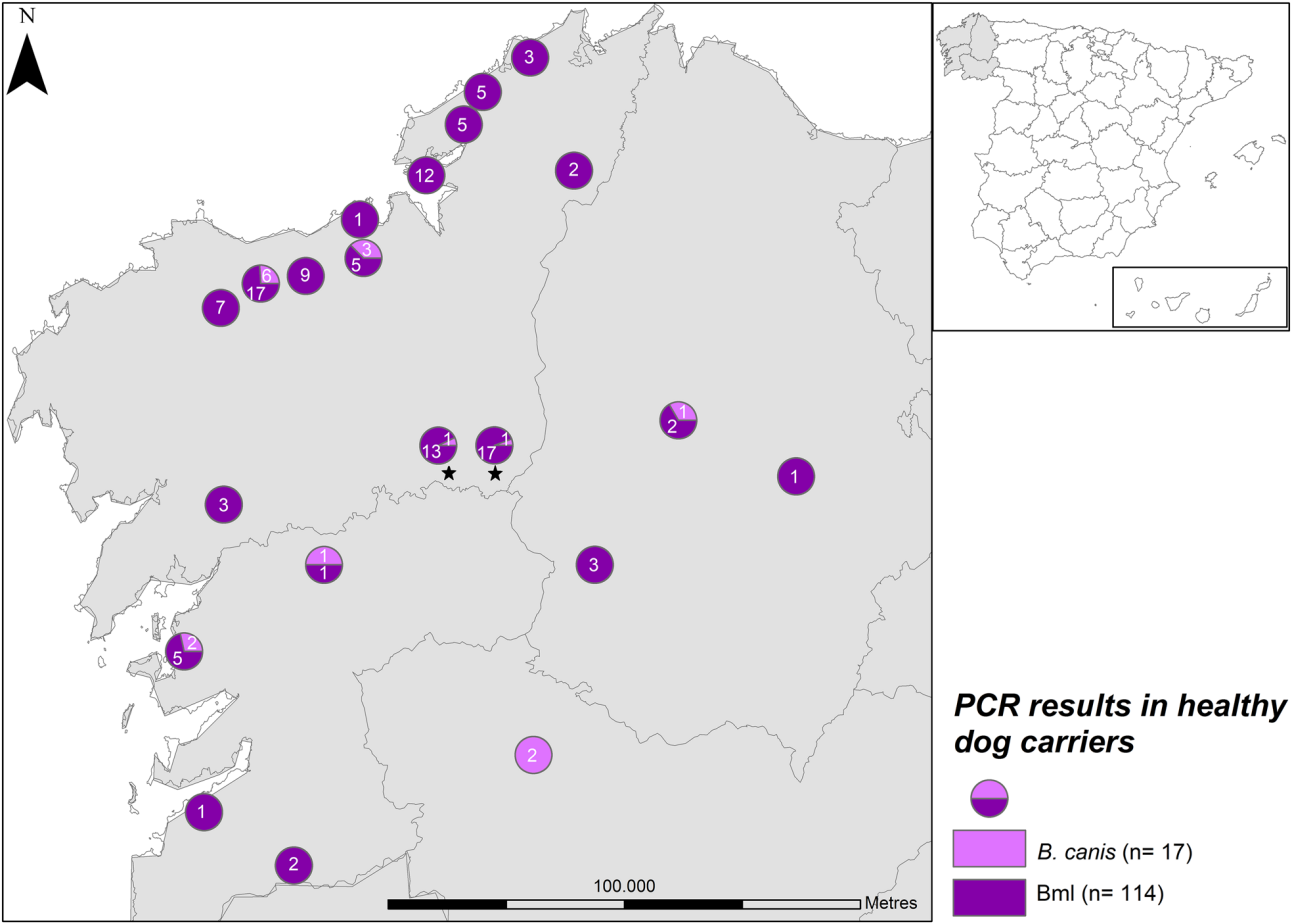
Geographical distribution of *Babesia* spp. infection in healthy dog carriers in northwestern Spain (Galicia). Stars indicate co-infection with *B. canis* and Bml

The overall prevalence of piroplasm infection in healthy Galician dogs was estimated at 17.1% (129/756) by PCR-RFLP. By species, prevalences were 2.2% (17/756) for *B. canis* and 15.1% (114/756) for Bml. Co-infection with *B. canis* and Bml was identified in 2 (0.3%) dogs. Higher prevalences were detected in hunting dogs for Bml at 25.5% (85/333) and for *B. canis* in stray dogs at 6.6% (14/211). Molecular prevalences (as determined by PCR-RFLP and Bml-specific PCR) according to geographical distribution and dog population surveyed are provided in Fig. 2.

All positive PCR-RFLP results for Bml were in agreement with positive Bml-specific PCR results. In addition, two samples testing positive for *B. canis* by nested-PCR and PCR-RFLP were also positive by Bml-specific PCR, confirming *B. canis* and Bml co-infection.

### Microscopy results

Intraerythrocytic forms consistent with piroplasm merozoites were observed by light microscopy in 26 out 756 blood samples (3.4%), of which 2 were compatible with large piroplasms and 24 were compatible with small piroplasms. Out of 26 positive blood smears, 3 (0.4%) and 23 (3%) were from stray and hunting dogs, respectively. Co-infections were not detected in smears. All positive blood smears showed mild parasitaemia and were PCR positive. However, 102 negative blood smears were PCR-positive for piroplasm infection. There was fair agreement between these two diagnostic tools (kappa value of 0.29).

### Epidemiological data on Bml-infected healthy dogs

In Table 1 we provide epidemiological data for the 114 confirmed cases of Bml. Among the 756 dogs included in this study, 333 were hunting dogs, of which 85 tested positive for Bml infection (25.5%). Significant differences were detected for hunting dogs *versus* stray dogs (11.4%, 24/211) or pets (2.4%, 5/212) (*χ*^2^ = 57.42, *df* = 2, *P *< 0.0001). Among dogs living in kennels, higher percentages of Bml positive were observed (26.9%), this link being significant (*χ*^2^ = 66.18, *df* = 4, *P *< 0.0001).

**Table 1:** Epidemiological variables recorded in 114 Bml-infected healthy dogs (PCR and sequencing confirmed)

Variable		*n*	Positive Bml (%)	*χ*^2^-value	*df*	*P*-value
Dog population	Hunting dog	333	85 (25.5)*	57.429	2	≤0.0001
	Pet	212	5 (2.4)			
	Stray dog	211	24 (11.4)			
Study area	A Coruña	285	99 (34.7)*	139,734	3	≤0.0001
	Lugo	165	6 (3.6)			
	Ourense	136	0			
	Pontevedra	170	9 (5.3)			
Breed	Crossbreed	371	63 (17)	2016	1	0.156
	Pure breed	377	50 (13.3)			
Age (years)	< 3	202	27 (13.4)	0.885	2	0.642
	3–5	248	41 (16.5)			
	>5	264	41 (15.5)			
Size (kg)	≤5	27	5 (18.5)	23.876	3	≤0.0001
	>5 ≤ 10	110	25 (22.7)*			
	> 10 ≤ 25	333	61(18.3)*			
	≥25	228	13 (5.7)			
Sex	Non-neutered male	255	54 (21.2)*	9.871	3	0.02
	Neutered male	66	5 (7.6)			
	Non-sterilized female	235	40 (17)			
	Sterilized female	78	8 (10.3)			
Habitat	House with garden	142	1 (0.7)	66.180	4	≤0.0001
	Flat	62	3 (4.8)			
	Kennel	316	85 (26.9)*			
	Farm	21	1 (4.8)			
	Shelter	211	24 (11.4)			
Fox-hunting	Yes	140	44 (31.4)*	10.974	1	0.001
	No	152	23 (15.1)			
Ticks	Yes	42	10 (23.8)	1.988	1	0.165
	No	607	95 (15.7)			
Ectoparasiticides	Yes	601	99 (16.5)	0.231	1	0.631
	No	75	14 (18.7)			
Clinical signs^a^	Presence	92	7 (7.6)	4.709	1	0.03
	Absence	657	107 (16.3)*			
Body condition	Thin	93	26 (28)*	20.767	2	≤0.0001
	Normal	463	83 (17.9)			
	Overweight	85	2 (2.4)			

* Significant differences observed

^a^Some clinical signs observed but not suggestive of acute canine piroplasmosis

By geographical region, dogs testing Bml-positive were distributed mainly across the northwestern coast (A Coruña Province) (Fig. 2). Thus, significant differences appeared between A Coruña Province and the other three provinces surveyed (*χ*^2^ = 139.73, *df* = 3, *P *< 0.0001), the prevalence of Bml being greatest in dogs from the northwestern coast of the region (34.7%; 99/285), followed by dogs from the southwestern coast (Pontevedra Province; 5.3%, 9/170) and dogs from northeastern Galicia (Lugo Province; 3.6%, 6/165). No Bml-infected healthy dogs were detected in the Ourense Province.

There were no differences related to age, breed or tick infestation; yet a greater number of positive cases (21.2%) were detected in non-neutered males (*χ*^2^ = 9.87, *df* = 3, *P* = 0.017). Significant correlations were also observed between Bml infection and a medium dog size (> 5 and ≤ 25 kg) (*χ*^2^ = 23.87, *df* = 3, *P *< 0.0001). Moreover, 28% (26/93) of dogs with a thin body condition were Bml-infected. Only 6.5% (42/649) of dogs surveyed were found to have ticks (data recorded during signalment). In addition, 23.8% (10/42) of dogs with ticks were infected with Bml, but no significance was detected for this risk factor. However, 88.9% of the dogs sampled had received ectoparasiticides (601/676).

Upon physical examination, 130 ticks were collected only from 18 hunting and three pet dogs. Of these, 0.8% (1/130) were larvae, 23.8% (31/130) were nymphs, 55.4% (72/130) were adult females and 20% (26/130) were adult males. These were subsequently identified as *R. sanguineus* (*s.l.*) (49.2%; 64/130), *I. hexagonus* (38.5%; 50/130), *I. ricinus* (6.9%; 9/130) and *Dermacentor reticulatus* (5.4%, 7/130). The most prevalent tick infesting Bml-positive dogs was *I. hexagonus* (59%, 13/22), followed by *D. reticulatus* (27.3%, 6/22) and *I. ricinus* (13.6%, 3/22).

**Fig. 3:**
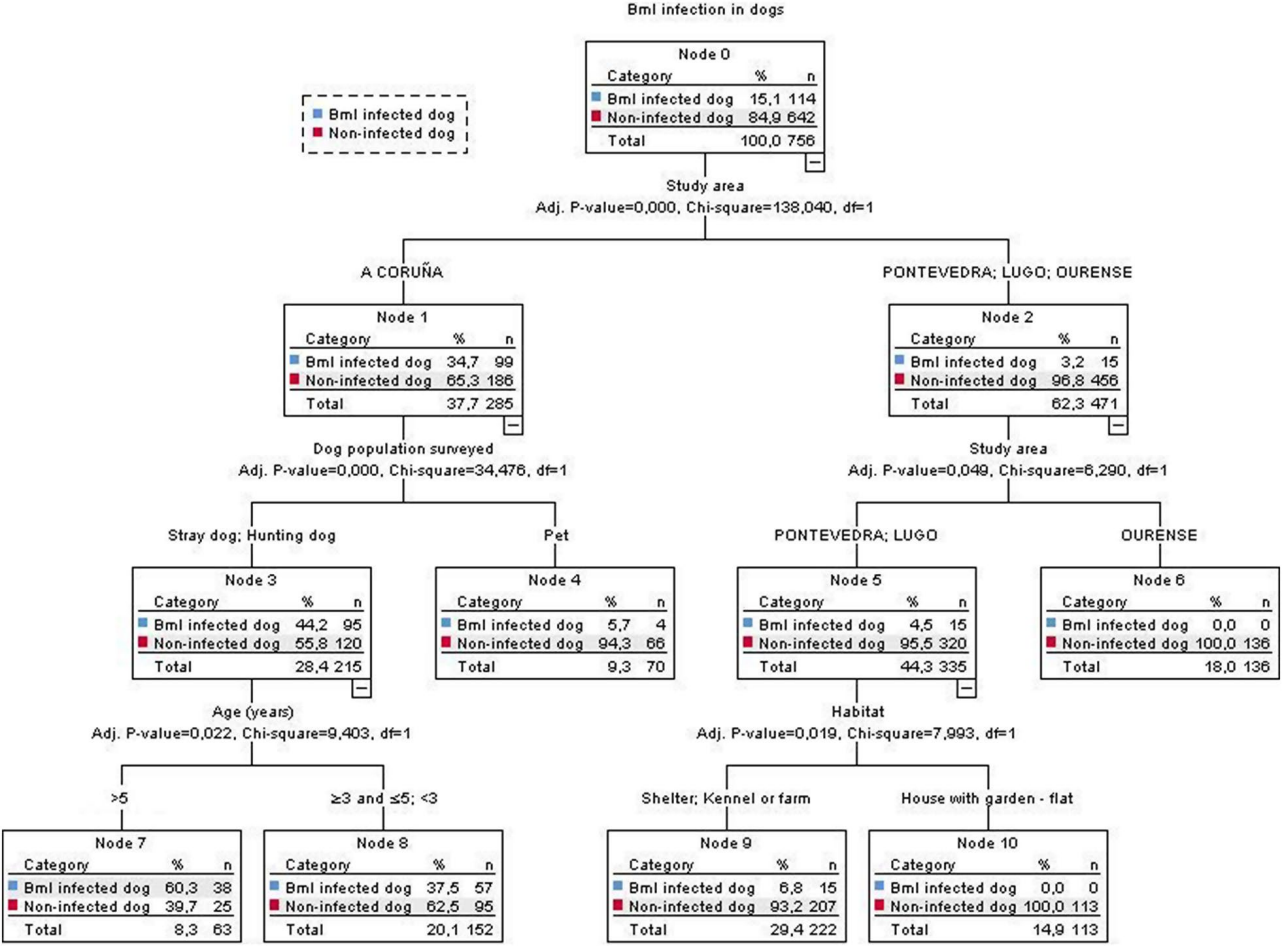
Decision tree model of Bml infection in the studied population. The decision tree model consists of predictors chosen according to their statistical significance, thereby allowing the detection of interactions with the previously selected variable (PCR-confirmed Bml-infected dogs). There are six terminal nodes that show a higher risk of infection with Bml according to the study area, dog population studied, age (years) and habitat (independent variables). Nodes 7 and 8 indicate a higher risk of Bml infection and nodes 4, 6, 9 and 10 a lower risk of Bml infection

To define subgroups of dogs that might show more than one type of condition or characteristic, a decision tree model was generated using the presence or absence of Bml infection as the dependent variable (Fig. 3). In this model, it may be observed that dogs showed a higher risk of Bml infection if they were living in A Coruña Province compared to the other three provinces of Galicia. Furthermore, if living in A Coruña, a dog was more likely to have Bml infection if it was a stray or hunting dog rather than a pet. Moreover, this risk increased when stray or hunting dogs were older than 5 years. Thus, hunting or stray dogs older than 5 years from A Coruña Province (terminal node 7 of the tree model) had a 60% risk of Bml infection, which was 4 times the overall Bml prevalence (node 0).

### Clinical status of Bml-infected healthy dogs

In the physical examination of all dogs, most showed no clinical signs (87.7%; 657/749) while 12.3% (92/749) did show some signs (not compatible with piroplasmosis) such as mild conjunctivitis or wounds due to the lifestyle of hunting and/or stray dogs. A total of 16.3% of asymptomatic dogs were infected with Bml while this occurred in only 7.6% of dogs showing some clinical sign.

Of the 114 Bml-infected dogs identified, 51 (46 hunting dogs, three stray dogs and two pets) were subjected to a CBC and biochemical profile after the diagnostic procedures. We were not able to collect a sufficient number of fresh blood samples for CBC and biochemical profiles in all Bml-infected dogs.

Four of these dogs (4/51) had been treated previously for canine babesiosis but none showed clinical signs suggesting canine piroplasmosis at the time of sampling. Only two dogs that died due to renal failure after the diagnosis of Bml were reported. Only one of these two dogs was examined (CBC, biochemical profile and urinalysis) immediately after diagnosis during a hunting season (October). This animal showed acute renal failure with non-regenerative anaemia, elevated creatinine, urea and SDMA. Urinalysis revealed bilirubin in urine with a normal urine gravity (1022) and non proteinuric (UPC ratio 0.16). This dog could not be monitored due to its owner’s availability. Finally, the owner decided euthanasia.

The 50 remaining dogs were asymptomatic. Clinicopathological findings in the 51 Bml-infected but healthy dogs are provided in Table 2. Haematological findings were slight regenerative anaemia in 18.75% (9/51) and non-regenerative anaemia in 8.33% (4/51). Mean haemoglobin concentrations, red blood cell counts and haematocrits were, nevertheless, clearly higher than equivalent values described for a group of diseased infected dogs by Miró et al. [4]. Furthermore, MCV values were slightly higher and MCHC values slightly lower in the healthy infected dogs compared to reference values. Biochemical profiles revealed slightly elevated SDMA and total protein values compared to reference values.

**Table 2:** Clinicopathological findings in 51 Bml-infected healthy dogs compared to Bml-infected sick dogs

Blood parameter (reference interval)	Group	*n*	Mean ± SD	95% CI	Percentile			*P*-value
					25th	50th	75th	
Erythrocytes (5.50–8.50) × 10^6^/µl	Healthy	51	5.21 ± 1.20	4.87–5.55	4.33	5.66	6.05	≤0.0001
	Sick^a^	72	3.28 ± 1.63	2.89–3.66	2.16	2.77	4.32	
Haematocrit (37.00–55.0)%	Healthy	51	42.80 ± 11.13	39.67–45.93	34.90	43.40	49.30	≤0.0001
	Sick^a^	72	27.13 ± 11.91	24.32–29.92	18.60	23.45	36.20	
Haemoglobin (12.00–18.00) g/dl	Healthy	51	12.01 ± 2.83	11.21–12.80	9.80	12.70	13.90	≤0.0001
	Sick^a^	72	7.76 ± 3.75	6.87–8.64	5.00	6.50	10.20	
MCV (60.00–76.00) fl	Healthy	51	80.89 ± 8.85	78.40–83.38	75.80	82.20	86.80	0.495
	Sick^a^	71	82.42 (8.25)	80.46–84.38	76.00	81.50	87.30	
MCHC (32.00–36.00) g/dl	Healthy	51	28.80 ± 3.08	27.93–29.67	26.90	28.50	29.80	0.456
	Sick^a^	71	28.26 ± 2.61	27.64–28.88	26.70	28.20	29.70	
Leukocytes (6–17) × 10^3^/µl	Healthy	51	11.55 ± 3.86	10.46–12.63	9.09	10.71	13.87	0.006
	Sick^a^	72	14.42 ± 6.26	12.95–15.89	10.50	13.32	17.10	
Platelets (200–500) × 10^3^/µl	Healthy	51	207.70 ± 105.76	177.96–237.45	147.00	192.00	251.00	0.021
	Sick^a^	59	158.46 ± 90.35	134.90–182.00	90.00	165.00	218.00	
Urea (21–59) mg/dl	Healthy	51	41.01 ± 28.41	33.02–49.01	28.00	35.00	43.00	0.384
	Sick^a^	71	60.49 ± 90.34	39.11–81.88	28.00	37.00	47.00	
Creatinine (0.5–1.5) mg/dl	Healthy	51	0.70 ± 0.35	0.60–0.80	0.50	0.60	0.80	0.255
	Sick^a^	71	0.95 ± 1.35	0.63–1.27	28.00	37.00	47.00	
Total proteins (4.8–7.8) g/dl	Healthy	51	7.90 ± 1.06	7.60–8.20	7.20	7.80	8.30	≤0.0001
	Sick^a^	71	6.93 ± 1.09	6.67–7.19	6.20	6.90	7.80	
ALT (26–89) UI/l	Healthy	51	51.76 ± 56.50	35.90–67.65	26.00	37.00	55.00	0.006
	Sick^a^	70	48.34 ± 110.43	22.01–74.67	20.00	28.00	42.00	
AST (16–89) UI/l	Healthy	51	42 ± 13.82	37.99–46.01	32.00	39.00	48.00	–
SDMA (0–14) µ/dl	Healthy	48	15.42 ± 5.41	13.84–16.99	12.00	15.00	18.00	–

*Abbreviations*: CI, confidence interval; SD, standard deviation

^a^Data reported previously by Miró et al. [9]

## Discussion

Studies in European countries, including some in Spain, have focused on the seroprevalence of canine infection with *Babesia* spp. and on PCR-based prevalences. One of the first surveys conducted by Camacho et al. [37] detected a 1.9% prevalence of Bml in dogs in Galicia by microscopy (later confirmed by PCR but only in dogs in which Bml was initially suspected by microscopy) [37]. The overall prevalence as determined by microscopy in our study was 3.2%, which is higher than the prevalence reported by Camacho et al. [37]. In a later study in northwestern Spain, a 62.5% prevalence was found in dogs with clinical signs consistent with canine piroplasmosis whose blood samples were analysed by PCR [4]. In a study conducted in Serbia, an overall prevalence of 10.1% Bml infection was detected in 158 healthy dogs [14]. In France, 0.7% of dogs were infected with Bml [13]. In the present study, the overall molecular prevalence of Bml infection was 15.1% in healthy dogs in Galicia, which is lower than that observed in an earlier study in sick dogs in Spain (62.5%), but higher than that obtained in earlier studies in healthy dogs in Europe (0.1%, 0.7% and 10.1% in Croatia, France and Serbia, respectively).

The prevalence of Bml infection reported for dogs in Serbia is lower than that described in studies carried out in foxes from neighbouring countries such as Hungary [22] and Bosnia and Herzegovina [27]. In Spain, the highest prevalence of Bml infection has been reported in red foxes in the north-west (72%), specifically in red foxes from northeastern Galicia (Lugo Province) [15]. However, in the present study, the highest Bml prevalences detected were in dogs from the northwestern coast of Galicia (A Coruña Province) (34.7%). This could be because there were few sampled foxes from the hyperendemic regions of Bml on the northwestern coast of Galicia. In the study of canine babesiosis in Serbia, a significant difference was also established between Bml infection and geographical area [14]. Differences in *Babesia* spp. prevalences suggest possible local effects involving vector distribution, density, and differences in temporal evolution of life stages, all of which may condition the exposure of dogs to tick-borne diseases [43].

Consistent with previous surveys [15, 44], our results suggest that molecular techniques could help detect Bml infection in dogs compared to microscopy techniques. This may be especially true in animals with chronic disease showing low parasitaemia levels.

In this study, the healthy dog population from Galicia was grouped according to risk level into stray or hunting dogs and pets. The higher piroplasm prevalences were detected in 25.52% of hunting and 6.63% of stray dogs for Bml and *B. canis*, respectively. It is assumed that stray dogs have a higher risk of tick exposure as they live outdoors. Stray dogs wander around with no preventive measures against ectoparasites, and hunting dogs frequently roam mountain and forest areas where the habitats of wild animals (e.g. red foxes) are found.

Our study reveals that hunting dogs in northwestern Spain are the most often infected with Bml. Thus, previous surveys in this same region indicate that hunting dogs show a higher risk of Bml infection than companion or guard dogs [4, 45]. These studies, however, did not provide prevalence data. Similar results have been observed in studies carried out in southern Italy and Romania, where seroprevalences of *B. vogeli* and *B. canis* infection in hunting dogs were higher [46, 47]. However, comparisons are hindered by diverse diagnostic methods, sample sizes, origins and study times. We selected the use of molecular tools for our study, which is the reference method for detecting this small piroplasm in healthy dog carriers.

The data emerging from our study indicate no significant differences in Bml infection by breed or age, as also described in the *B. canis* seroprevalence study conducted in Romania [47]. Reported frequencies for *Babesia* spp. infection have nevertheless been higher in younger sick dogs from endemic areas [4, 8]. According to our tree model, Bml infection prevalence was increased in older dogs (> 5 years), indicating age is a risk factor for Bml infection in hunting or stray dogs in the A Coruña Province. As suggested by other authors, this finding in healthy dogs in an endemic area of canine babesiosis probably reflects the long-term exposure to ticks rather than an enhanced susceptibility to *Babesia* infection [46]. However, young animals showed clinical signs more often explained by their first contact with the pathogen [4].

Our data point to higher proportions of infected male than female dogs, as described for Bml in foxes in the Great Britain [23] and for *B. vogeli* in dogs in Italy [46]. Furthermore, we detected a higher percentage of infected non-sterilized than sterilized animals. This could be because usually hunters do not like to sterilize their dogs. Vertical transmission has been reported for other small *Babesia* species, mainly *B. gibsoni* [48], and other protozoan pathogens showing a high prevalence in hunting dogs such as *Leishmania infantum* [49]. This route of transmission was also hypothesized for Bml in a 1–2-week-old puppy in Sweden [12], such that the high prevalence of Bml found here in non-sterilized hunting dogs could indicate this is a potential route of transmission. This issue requires confirmation in further studies. The links observed in our study between animal size (5–25 kg), body condition or a fox-hunting dog and Bml infection could reflect the fact that fox-hunting dogs are usually thin (e.g. beagle or griffon breeds) as proposed by Miró et al. [4]. In areas with a high density of red foxes such as northwestern Spain, hunting to control this wild carnivore is common practice (under current national legislation). Thus, hunting dogs and red foxes share habitats and often come into direct contact [15].

The four species of ticks found infesting dogs in the present study, *I. hexagonus*, *R. sanguineus* (*s.l.*), *D. reticulatus* and *I. ricinus*, are common in dogs in Spain [50]. However, we observed here that the most prevalent ticks collected in Bml-positive animals were adult stages of *I. hexagonus*, followed by *D. reticulatus* and *I. ricinus*. Consistently, the most abundant tick species observed in red foxes from northern and northwestern Spain have been immature stages of *I. hexagonus* [15, 51]. However, because of the small proportion of infested dogs in our study and in the absence of experimental infection studies, we cannot confirm their role as potential vectors of Bml. Other forms of transmission such as vertical, direct or mechanical cannot be discarded [52]. Future studies should address the different transmission routes of Bml among high-risk dogs in northwestern Spain.

*Babesia canis* and Bml were the only species found infecting healthy dogs in Galicia, the prevalence of Bml being higher than that of *B. canis* when determined by PCR. However, in Galician dogs showing clinical signs compatible with babesiosis, *B. canis* and Bml infection were detected by PCR in 27.5% and 22.5%, respectively [53]. In agreement with our study, the majority of Bml infections in sick dogs were detected in A Coruña Province while *B. canis* were mainly detected in the remaining provinces from Galicia [53]. *Babesia vogeli* has been frequently reported in dogs in southern Italy. Veneziano et al. [46] described that overall *B. vogeli* and *B. canis* PCR prevalences are lower than their overall seroprevalences in hunting dogs. Thus, in the present study, PCR prevalences were lower for *B. canis* than Bml suggesting than Bml infection is more prevalent than large piroplasm infection in healthy dogs. The maintenance of Bml infection in the healthy canine population could be favoured by the fact that Bml infection is refractory to current piroplasmosis treatment. Curative treatment is therefore difficult to document.

In a study performed in 269 Pit Bull dogs, anaemia was correlated with *B. gibsoni* infection (63% of infected dogs) [54]. In our study, only 25% of infected dogs showed mild anaemia. The mean haematological data obtained in healthy carrier dogs were mildly increased MCV and mildly decreased MCHC, but within anaemia (erythrocytes, haematocrit and haemoglobin means were normal). More severe CBC anomalies have been also reported by others in Bml-infected sick dogs with severe regenerative anaemia [4]. Biochemical profiles indicated slightly elevated SDMA. Old hunting Bml-infected dogs were described to have a higher risk of developing azotaemia [55], while a few cases of azotaemia (9.8%) were detected in acute canine babesiosis caused by Bml [4].

In the present study, a high number of healthy dog carriers of Bml were detected. Usually, carrier dogs with chronic babesiosis do not show clinical signs unless their health deteriorates as a result of immunosuppressive treatment, splenectomy or other immune-compromised circumstance [2]. Some authors have described subclinical or chronic infection states (detected by PCR) in a significant proportion of dog populations such as *B. gibsoni* in kennels housing American Pit Bull Terriers [52] and *B. vogeli* in Greyhounds [56].

Preventive measures in endemic areas of canine babesiosis for travelling dogs should incorporate *Babesia* spp. screening and monitoring anaemia in healthy stray or hunting dogs to avoid the spread of Bml infection to other regions.

## Conclusions

*Babesia canis* and Bml were the only species found infecting healthy dogs in Galicia, though unexpectedly, the prevalence of Bml was greater than that of *B. canis*. Factors correlated with a higher Bml infection risk were being a fox-hunting adult dog and living in the A Coruña Province. Although Bml seems to be widely distributed in red foxes in Galicia, clinically healthy but infected dogs are acting as subclinical carriers, which could contribute to the spread of Bml among dog populations all over Europe. Veterinary practitioners should undertake Bml screening and anaemia monitoring in those risk groups (both in endemic areas such as Galicia and/or travelling dogs) and should implement protocols to prevent its transmission in kennels and following adoptions. Healthy hunting dogs from endemic areas, especially those older than five years of age, should not be use as blood donors or for breeding because of a risk of Bml infection, unless they have been previously PCR screened.

## Abbreviations

Bml: *Babesia microti*-like piroplasm
CBC: complete blood count
WBC: white blood cell count
RBC: red blood cell count
MCH: mean corpuscular haemoglobin
MCHC: mean corpuscular haemoglobin concentration
MCV: mean corpuscular volume
RDW: red cell distribution with
AST: aspartate aminotrasnsferase
ALT: alanine aminotransferase
SDMA: symmetric dimethylarginine
EDTA: ethylene diamine tetra-acetic acid
PCR: polymerase chain reaction
RFLP: restriction fragment length polymorphism
UPC: urinary protein creatinine ratio
